# Preparation of Antioxidant Peptide by Microwave- Assisted Hydrolysis of Collagen and Its Protective Effect Against H_2_O_2_-Induced Damage of RAW264.7 Cells

**DOI:** 10.3390/md17110642

**Published:** 2019-11-14

**Authors:** Yan Li, Jie Li, Sai-Jun Lin, Zui-Su Yang, Huo-Xi Jin

**Affiliations:** 1 Zhejiang Provincial Engineering Technology Research Center of Marine Biomedical Products; School of Food and Pharmacy, Zhejiang Ocean University, Zhoushan 316022, China; m18625623399@163.com (Y.L.); lijie1749@126.com (J.L.); abc1967@126.com (Z.-S.Y.); 2 Hangzhou Institute for Food and Drug Control, Hangzhou 310052, China; saijunlin@126.com

**Keywords:** *Acaudina molpadioides*, antioxidant peptides, microwave-assisted hydrolysis, oxidative stress protection

## Abstract

Antioxidant peptides have elicited interest for the versatility of their use in the food and pharmaceutical industry. In the current study, antioxidant peptides were prepared by microwave-assisted alkaline protease hydrolysis of collagen from sea cucumber (*Acaudina molpadioides*). The results showed that microwave irradiation significantly improved the degree of hydrolysis of collagen and the hydroxyl radical (OH⋅) scavenging activity of hydrolysate. The content and OH⋅ scavenging activity of collagen peptides with molecular weight ≤ 1 kDa (CP_S_) in the hydrolysate obtained at 250 W increased significantly compared with the non-microwave-assisted control. CP_S_ could scavenge OH⋅ and 2,2-diphenyl-1-picrylhydrazyl (DPPH) radical in a dose-dependent manner. The scavenging activity of OH⋅ and DPPH radical was 93.1% and 41.2%, respectively, at CP_S_ concentration of 1 mg/mL. CP_S_ could significantly promote RAW264.7 cell proliferation and reduce the Reactive Oxygen Species (ROS) level of H_2_O_2_-induced damage in RAW264.7 cells in a dose-dependent manner. Furthermore, all CP_S_-treated groups exhibited an increase in superoxide dismutase (SOD) and glutathione peroxidase (GSH-Px) and a decrease in malondialdehyde (MDA) level compared with the control. These results showed that CP_S_ could effectively protect RAW264.7 cells from H_2_O_2_-induced damage, implying the potential utilization of CP_S_ as a natural antioxidant for food and pharmaceutical applications.

## 1. Introduction

Bioactive peptides are short peptides with some bioactive functions, such as antioxidant, antimicrobial, antitumor, or antihypertensive activity. Bioactive peptides have been widely used in health-food, nutraceuticals, and pharmaceutical preparations due to their significant biological functions and efficient absorption [1]. As one of several promising bioactive peptides, antioxidant peptides have become a topic of great interest because of the association between many human diseases and oxidative stress [2]. When humans are subjected to oxidative stress, large amounts of reactive oxygen species (ROS) are produced. Abundant quantities of ROS (superoxide, hydrogen peroxide, and hydroxyl) tend to react with biological molecules, such as protein and DNA, often leading to liver disease, heart disease, and cancer because of oxidative damage of cells [3,4]. ROS-induced oxidative cell damage is usually accompanied by an increase in lipid peroxides [5]. It was known that malondialdehyde (MDA) is the major secondary metabolite of lipid peroxidation induced by ROS. The content of MDA is a marker of lipid peroxidation and the degree of cell damage [6]. The superoxidase dismutase (SOD) and glutathione peroxidase (GSH-Px) are the major antioxidant enzymes, which play a key role in protecting cells from oxidative damage [7]. Antioxidants can protect the body from damage by removing excess ROS, reducing the MDA, and improving the activities of SOD and GSH-Px. Therefore, as a natural antioxidant, antioxidant peptides have great potential of utilization in pharmaceutical industries.

At present, antioxidant peptides are mainly obtained by separation and purification directly from the plant or animal tissues, chemical synthesis, microbial fermentation, or enzymatic hydrolysis of protein. However, the separation and purification of antioxidant peptides directly from the plant or animal tissues, and fermentation products are expensive. Chemical synthesis of peptides also requires sophisticated and expensive equipment and reagents, and the chemical reagents involved may be harmful to humans and/or cause environmental pollution. Therefore, enzymatic digestion of protein has been the most common method for preparation of antioxidant peptides due to the simplicity, safety, low-cost, and sustainability of the approach [8]. Collagen is an important structural protein rich in hydroxyproline, which has been employed in food and medical applications for decades with excellent biocompatibility and safety [9,10]. For example, the collagens from echinoderms (sea urchin, starfish, and sea cucumber) can be used to develop collagen barrier-membranes for guided tissue regeneration due to the good mechanical performances [11]. However, the large molecular weight of the compound creates barriers in absorption and utilization in the body. Therefore, collagen peptides, smaller proteins hydrolyzed from collagen, have received increasing attention because of their physicochemical properties and biological activity [12,13,14]. However, one of the limitations of the collagen peptides production process is the length of time required in conventional hydrolysis methods due to its dense fiber structure [8]. This limitation can potentially be overcome by using microwave assisted hydrolysis. Studies have shown that microwaves cause different biological and chemical effects depending, among other factors, upon strength, frequencies and length of exposure [15]. Microwave radiation penetrates into the interior of the protein molecule, exposing the hidden enzymatic sites and enabling a more thorough hydrolysis [16]. Microwave-assisted enzymatic hydrolysis of protein can, therefore, significantly reduce the overall hydrolysis time and increase the yield of peptide by intensifying molecular movements and collisions [8]. Previous studies had also demonstrated microwave-assisted enzymatic hydrolysis to be one of the most effective methods to prepare peptides [8,17,18].

Sea cucumbers, of which more than 1400 species exist around the world, are important marine resources of protein containing high amounts of collagen in their body wall [19]. Several researchers have reported the development and characteristics of various collagens from sea cucumbers [20,21,22,23]. The common sea cucumber, *Acaudina molpadioides,* is widely distributed in East China Sea, especially in the coastal areas of Ningbo and Zhoushan. Traditionally, due to the lack of key technologies for industrialization, *Acaudina molpadioides* resources have not been utilized to their potential and continue to sell for a low price. The preparation of collagen peptides using *Acaudina molpadioides* has great potential due to the good biological function of collagen peptides and inexpensive raw materials of *Acaudina molpadioides* [24]. Studies have shown that collagen peptides of sea cucumber have a demonstrated ability to scavenge free radicals and other peroxides [25]. In previous studies, we had prepared an antioxidant peptide from collagen of *Acaudina molpadioides* (ASC-Am) by microwave-assisted neutral protease hydrolysis [26]. Different proteases hydrolyze different sites of the protein to produce peptides of different amino acid sequences. Therefore, in this study, we focus on production of antioxidant peptides from ASC-Am by microwave-assisted alkaline protease hydrolysis. Furthermore, the protective effect of antioxidant peptides against peroxide (H_2_O_2_)- induced damage of RAW264.7 cells was investigated by measuring cell proliferation, ROS, MDA, SOD, and GSH-Px levels.

## 2. Results and Discussion

### 2.1. Effect of Microwave Assisted on Alkaline Protease Hydrolysis of ASC-Am

**Figure 1:**
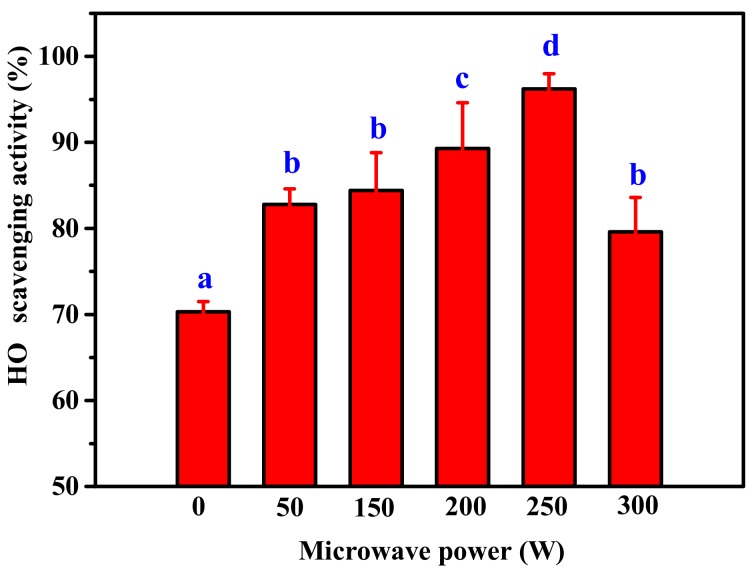
Effect of microwave power on OH· scavenging activity of collagen hydrolysate. The concentration of hydrolyzed product was 2 mg/mL. Values with different letters are significantly different (*p* < 0.05).

The effect of microwave power on OH· scavenging activity of hydrolysate by alkaline protease digestion of ASC-Am was investigated (Figure 1). The results demonstrated that OH· scavenging activities were significantly improved at all tested microwave powers (50–300 W) as compared to non-microwave assisted hydrolysis. The enhanced OH· scavenging activity when the microwave power increased from 50 to 250 W suggested that the higher microwave power enabled the higher probability of contact between ASC-Am and alkaline protease, resulting in more small peptides with antioxidant activity. However, excessive molecular collisions cause the denaturation of alkaline protease, which may make the OH· scavenging activity begin to decrease when the microwave power continues to increase to 300 W. The maximum OH· scavenging activity was observed at 250 W of microwave power, which increased from 70.3% in non-microwave assisted to 96.2% in this treatment. Therefore, 250 W was selected as the optimal power for microwave-assisted alkaline protease digestion of ASC-Am.

In addition, we investigated the content and molecular weight distribution of the peptides in hydrolyzate under microwave assisted digestion with 250 W. As shown in Table 1, compared with 0 W, the total content of CP_L_ at 250 W decreased, but the content of CP_M_ and CP_S_ increased by 48% and 30.2%, respectively. Furthermore, the proportions of CP_M_ and CP_S_ in hydrolyzate obtained at 250 W were both higher than those at 0 W. These results suggested that microwave assisted hydrolysis can significantly promote the breakdown of ASC-Am into smaller molecular weight peptides. The results of this study were similar to those of Uluko et al. (2015), which showed that the hydrolyzed product under microwave irradiation increased by 184.9% compared to the control [27].

**Figure 2:**
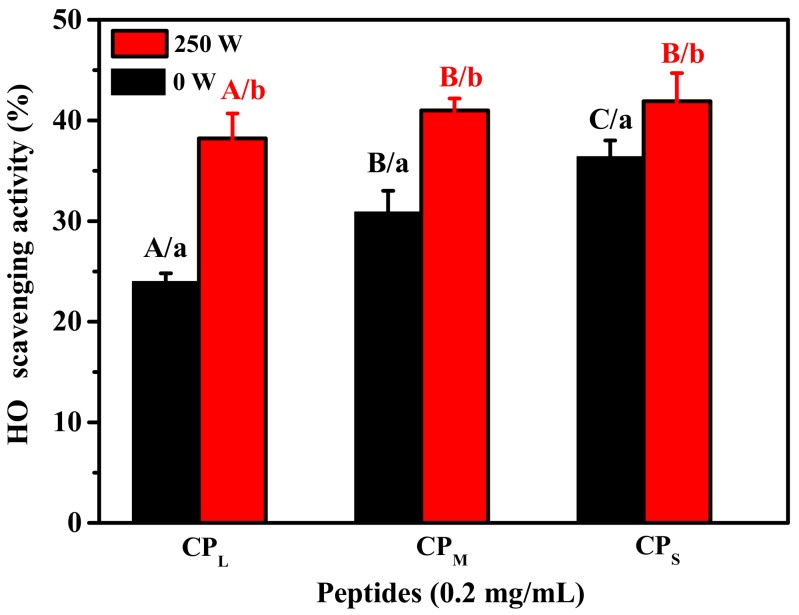
The OH· scavenging activity of peptides with different Mw (CP_L_, Mw > 5 kDa; CP_M_, 1 kDa < Mw ≤ 5 kDa; CP_S_, Mw ≤ 1 kDa) from hydrolysate of ASC-Am. (a–b) Values with different letters indicated significant differences in the same samples at different microwave powers (*P* < 0.05). (A–C) Values with different letters indicated significant differences in different samples at the same microwave powers (*p* < 0.05).

The molecular weight of peptides has a significant effect on the antioxidant activity [28]. It was evident that the peptides with smaller molecular weight revealed a stronger antioxidant activity (Figure 2), which was similar to the results of previous reports [29]. In addition, compared with those under 0 W of microwave power, the OH· scavenging activities of CP_L_, CP_M_, and CP_S_ under 250 W power were significantly improved. The reasons for this result may be: (1) microwave radiation probably exposed more cleavage sites of collagen for alkaline protease hydrolysis, resulting in an improvement in antioxidant activity of the peptides due to altered amino acid sequences of peptides; (2) the microwaves probably altered the amino acid sequence of the alkaline protease, resulting in a change in the cleavage site of alkaline protease.

### 2.2. Antioxidant Activity of CP_S_

**Figure 3:**
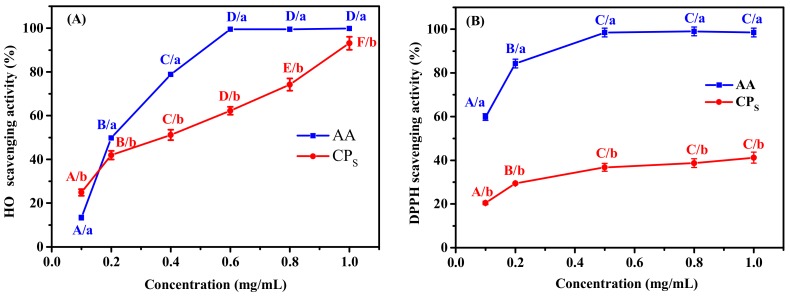
The scavenging activity of OH· (**A**) and DPPH (**B**) of CP_S_ obtained by alkaline protease hydrolysis of ASC-Am at 250 W of microwave power. Ascorbic acid (AA) was used as a positive control. (a–b) Values with different letters indicated significant differences in different samples at the same concentrations (*p* < 0.05). (A–F) Values with different letters indicated significant differences in the same samples at the different concentrations (*p* < 0.05).

In order to better investigate its antioxidant properties, the DPPH and OH· scavenging assays at different concentration of CP_S_ were studied and compared with the positive controls containing ascorbic acid (AA). The CP_S_ The OH· scavenging activity of CP_S_ increased from 24.9% at 0.1 mg/mL to 93.1% at 1.0 mg/mL (Figure 3A), indicating it scavenged OH· in a concentration-dependent manner. The EC_50_ value of CP_S_ was 0.4 mg/mL for OH·, which was lower than that of the peptide BSH-III (Mw ≤ 1 kDa) from protein hydrolysate obtained from bluefin leatherjacket skin (IC_50_ of 0.746 mg/mL) [30]. It is known that OH· can have a destructive effect on many biological macromolecules, such as proteins and nucleic acids; therefore, the high OH· scavenging activity of CP_S_ suggested the potential utilization of CP_S_ as a natural antioxidant for reducing or eliminating damage induced by OH· in food and pharmaceutical applications.

DPPH radicals are often used in antioxidant experiments because of their high stability [31]. The mechanism of DPPH assay is based on the reduction of DPPH solution in the presence of a hydrogen donor, leading to the formation of the non-radical form DPPH-H [32]. The scavenging of DPPH radicals by CP_S_ was well correlated with the concentration of CP_S_ (Figure 3B). The DPPH scavenging activity of CP_S_ was found to be 20.5% at a concentration of 0.1 mg/mL, which increased to 41.2% at 1.0 mg/mL, still much lower than that of AA. However, the DPPH scavenging activity of CP_S_ was significantly higher than that of the protein hydrolysate fraction (Mw < 1 kDa) from bluefin leatherjacket skin and skate cartilage [30,33].

### 2.3. Antioxidant Activity Evaluated by H_2_O_2_-Induced Injury Cell Model

#### 2.3.1. The Effect of H_2_O_2_ and CPs on the Proliferation of RAW264.7 Cells

**Figure 4:**
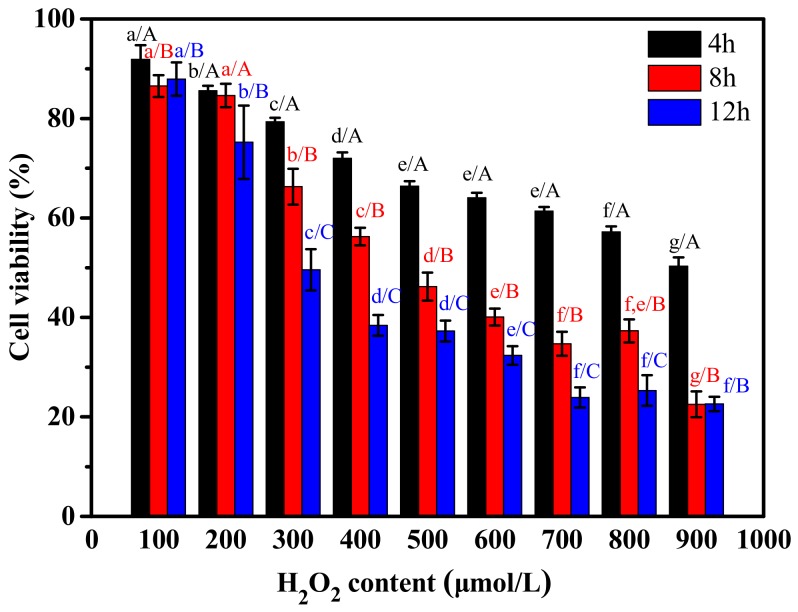
The effect of H_2_O_2_ on the viability of RAW264.7 cells. (a–g). Values with different letters indicated significant differences in the same time at different concentrations of H_2_O_2_ (*p* < 0.05). (A–C) Values with different letters indicated significant differences in the same concentrations of H_2_O_2_ at different times (*p* < 0.05).

To investigate the effect of H_2_O_2_ on viability of RAW264.7 cells, they were treated with different concentrations of H_2_O_2_. RAW264.7 cells revealed a significant decrease in viability with increasing concentration of H_2_O_2_ and treatment time (Figure 4). When the concentration of H_2_O_2_ was greater than 300 μM and the treatment time was longer than 8 h, the cell viability was significantly inhibited. Cell viability exposed to 500 µmol/L H_2_O_2_ for 8 h was 46.2% of the control value. In the subsequent experiments, the RAW264.7 cells were treated with 500 µmol/L H_2_O_2_ for 8 h to study the effect of CP_S_ on H_2_O_2_-induced injury.

**Figure 5:**
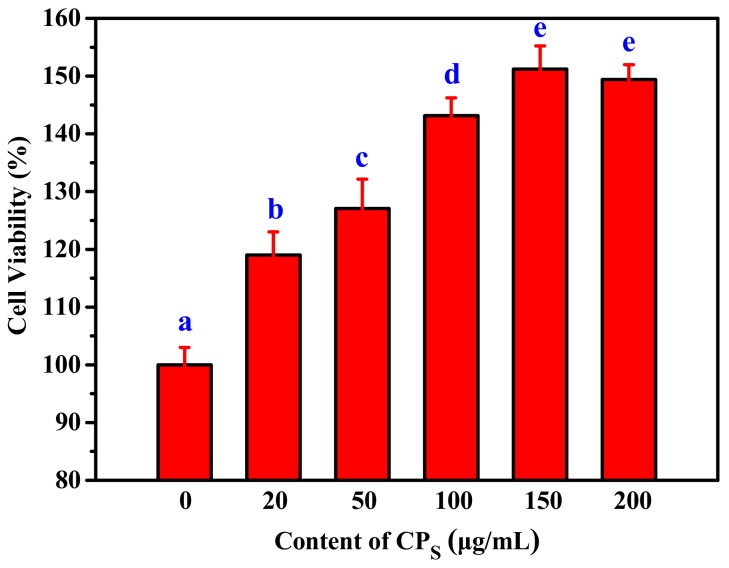
Effect of CP_S_ on viability of RAW264.7 cells. Values with different letters are significantly different (*p* < 0.05).

The effect of CP_S_-treated on the proliferation of RAW264.7 cells was shown in Figure 5. The viability of RAW264.7 cells in CP_S_ treatment group was significantly improved compared to the control group when the concentration of CP_S_ increased from 20 µg/mL to 150 µg/mL (Figure 5). This demonstrated that CP_S_ could effectively promote the proliferation of RAW264.7 cells in a dose-dependent manner. However, a significant increase in cell viability was not observed when cells were treated with 200 µg/mL. Therefore, 100 µg/mL, 150 µg/mL and 200 µg/mL were selected as the low-, middle- and high-dose groups for subsequent experiments, respectively.

#### 2.3.2. Effects of CP_S_ on the ROS Levels in RAW264.7 Cells

**Figure 6:**
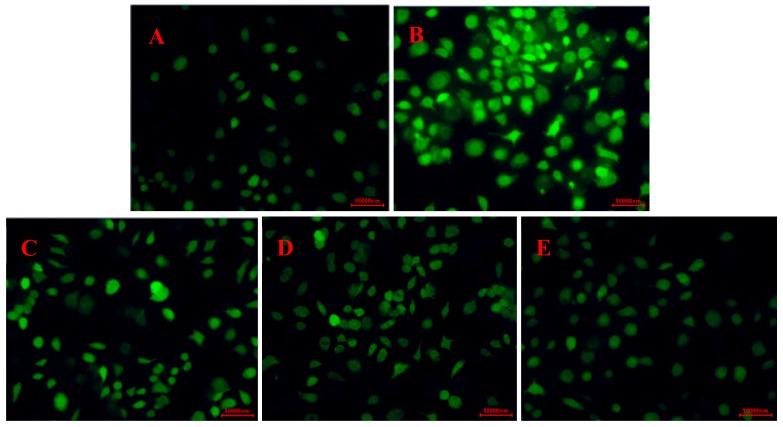
Effect of CP_S_ on ROS levels in RAW264.7 cells induced by H_2_O_2_. (**A)** Control group; (**B**) model group (500 µM H_2_O_2_); (**C**) low-dose group (100 µg/mL CP_S_ + 500 µM H_2_O_2_); (**D**) middle-dose group (150 µg/mL CP_S_ + 500 µM H_2_O_2_); (**E**) high-dose group (200 µg/mL CP_S_ + 500 µM H_2_O_2_).

In this section, the effect of CP_S_ on the ROS levels in RAW264.7 cells was investigated. The fluorescence intensity in RAW264.7 following H_2_O_2_ treatment was significantly larger than that of the control group (Figure 6). However, the addition of CP_S_ effectively reduced the ROS level relative to the model group, and the ROS level gradually decreased with the increase in CP_S_ concentration. The results suggested that the protective effects of CP_S_ against H_2_O_2_-induced injury of RAW264.7 cells may have resulted from inhibition of intracellular ROS production.

#### 2.3.3. Effect of CP_S_ on the Level of MDA, SOD, and GSH-Px in Cells

**Figure 7:**
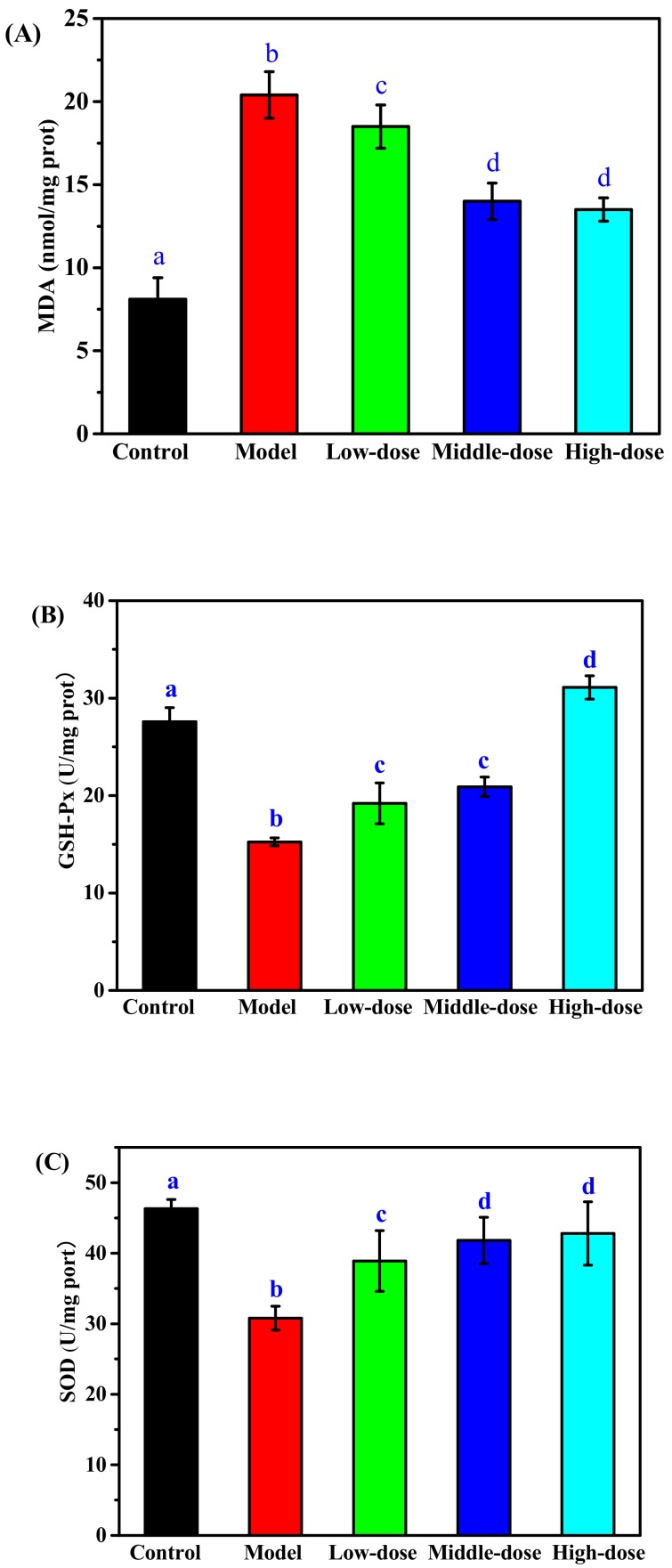
Effect of CP_S_ on levels of MDA (**A**), GSH-Px (**B**), and SOD (**C**) in RAW264.7 cells induced by H_2_O_2_. Model group (500 µM H_2_O_2_), low-dose group (100 µg/mL CP_S_ + 500 µM H_2_O_2_), middle-dose group (150 µg/mL CP_S_ + 500 µM H_2_O_2_), high-dose group (200 µg/mL CP_S_ + 500 µM H_2_O_2_). Values with different letters are significantly different (*p* < 0.05).

To evaluate the antioxidant activity of CP_S_, we investigated the effect of CP_S_-pretreated on the level of MDA, GSH-Px, and SOD respectively in H_2_O_2_-induced oxidative damage of RAW264.7 cells. The MDA level in the cells significantly increased following H_2_O_2_ treatment as compared to the control group (Figure 7A), indicating that the RAW264.7 cells were damaged by H_2_O_2_. A significantly lower MDA level compared to the control group was observed in the CP_S_-treated groups, especially in the middle- and high-dose group, indicating that CP_S_ decreased the oxidative damage level of cells caused by H_2_O_2_. Effects of CP_S_ on GSH-Px and SOD activity of RAW264.7 cells were shown in Figure 7B,C. The results showed that H_2_O_2_ caused a significant decrease in GSH-Px and SOD activity in RAW264.7 cells. Fortunately, the levels of GSH-Px and SOD were both markedly promoted in CP_S_-treated groups as compared to the control group. The group with the higher dose exhibited the highest level of GSH-Px, but the results between the low-dose and middle-dose groups were not statistically significant. The highest levels of SOD were observed in the high-dose and middle-dose groups, but the difference between them was not statistically significant. Qiu et al. had reported that collagen peptides could up-regulate the levels of SOD and GSH-Px, and down-regulate the contents of MDA, playing a protective role in antioxidant effects on Drosophila [34]. The collagen peptides from cod skin protected liver tissue against oxidative injure by increasing the activity of SOD and decreasing MDA [35]. Therefore, these results in this study demonstrated that collagen peptides from sea cucumber *Acaudina molpadioides* could protect RAW264.7 cells against H_2_O_2_-induced injury by inhibition of lipid peroxides and enhancement of antioxidant enzyme activity.

## 3. Materials and Methods 

### 3.1. Chemicals and Reagents

Sea cucumber *Acaudina molpadioides* was provided from Zhoushan Jingzhou Aquatic Food Co., Ltd., in Zhejiang Province of China. Fetal calf serum was provided by GIBCO (Invitrogen Corporation, Carlsbad, California, USA). The 2,2-dipehnyl-1-picryldydrazyl (DPPH), 3-(4,5- Dimethylthiazol-2-yl)-2,5-diphenyl tetrazolium bromide (MTT) and dimethyl sulfoxide (DMSO) were purchased from Sigma-Aldrich (Shanghai, China). Assay kits for the MDA, SOD, and GSH-Px were purchased from the Nanjing Jiancheng Bioengineering Institute (Nanjing, China). ROS detection kit was purchased from Beyotime Company (Jiangsu, China). All other reagents were analytical grade.

### 3.2. Preparation of Collagen Peptides

ASC-Am was prepared from the body wall of *Acaudina molpadioides* according to the method reported previously [26]. Two grams of ASC-Am were added into 200 mL buffer solution (pH 10.0) and pretreated with irradiation (0, 50, 150, 200, 250, and 300 W) for 30 minutes each in a microwave (XH-300A; Beijing Xianghu, China). Alkaline protease (≥200 U/mg, Shanghai Ryon Biological Technology Co., Ltd., Shanghai, China) was added to the sample for hydrolysis at 45 °C. After 1 h, the alkaline protease was inactivated in boiling water for 10 min. After centrifuging the hydrolysates at 10,000 rpm for 10 min, the supernatants were freeze-dried, and the scavenging activity of hydroxyl radical (OH⋅) was measured at the concentration of 0.2 mg/mL.

The hydrolysates obtained under the optimal microwave power were separated by ultrafiltration membranes with size exclusion of 5 kDa and 1 kDa. Three fractions (CP_S_, collagen peptides with Mw ≤ 1 kDa; CP_M_, collagen peptides with 1 kDa < Mw ≤ 5 kDa; and CP_L_, collagen peptides with Mw > 5 kDa) were collected and freeze-dried separately to measure the peptide content and scavenging activity of OH⋅ radical at the concentration of 0.2 mg/mL.

### 3.3. Antioxidant Activity Measurements

#### 3.3.1. Scavenging Activity of Hydroxyl Radical

The scavenging activity of OH⋅ of peptide was determined using the Fenton method [36]. Two mL peptide samples were mixed with 2 mL of phosphate buffer (0.2 M, pH 7.4), 1 mL of 1,10-phenanthroline (0.75 mM), 1 mL of FeSO_4_ (0.75 mM), and 1 mL of 0.3% H_2_O_2_ (v/v). The mixture was heated at 37 °C for 30 min, and the absorbance (A_1_) measured at 510 nm. The scavenging activity of OH⋅ was calculated using the following formula, where A_0_ is the absorbance of the blank with 2 mL of water instead of the peptide solution and A_2_ the absorbance of mixture with 1 mL of deionized water instead of the FeSO_4_ solution.

OH· scavenging activity (%) = [1−(A_1_ − A_2_)/A_0_] × 100% (1)

#### 3.3.2. Scavenging Activity of DPPH Radical

The scavenging activity of DPPH radical was tested according to previously reported methods [37]. The samples of peptides were dissolved in water with different concentrations 0.1, 0.2, 0.5, 0.8, and 1.0 mg/mL. Two mL of peptide samples were mixed with 2 mL of DPPH solution (0.2 mM) and 1 mL of ethanol. The mixture was incubated for 30 min at room temperature and then centrifuged at 5000 rpm for 5 min. The absorbance of the sample (A_s_) was measured at 517 nm using an ultraviolet-visible spectrophotometer. The scavenging activity of DPPH was calculated as shown in the formula:

DPPH scavenging activity (%) = [1−(A_s_ − A_b_)/A_c_] × 100% (2)

where A_c_ is the absorbance of the control group with 2 mL of water instead of the peptide sample; A_b_ is the absorbance of blank with 2 mL of ethanol in place of the DPPH solution.

### 3.4. The Effect of H_2_O_2_ and Peptides on the Proliferation of RAW264.7 Cells

RAW264.7 cells (purchased from Chinese Academy of Sciences) were grown in DMEM medium supplemented with 10% fetal bovine serum and incubated at 37 °C in a humidified atmosphere with 5% CO_2_. The peptides were dissolved in DMEM medium and diluted to different concentrations (20–200 µg/mL). Cell viability was determined using MTT assay. RAW264.7 cells were seeded in 96-well plates at 1 × 10^5^ cells/mL and incubated at 37 °C with 5% CO_2_ for 24 h. The cells were treated with different concentrations of H_2_O_2_ (100, 200, 300, 400, 500, 600, 700, 800, and 900 μmol/L) or peptides. A volume of 20 µL of MTT solution (2 mg/mL) was added to each well and incubated for 4 h. The supernatant was removed and 100 µL of Dimethyl sulfoxide (DMSO) was added into each well to dissolve the formazan crystals. The plates were shaken for 5 min to dissolve the crystals completely, and absorbance was measured at 490 nm. The cell viability with H_2_O_2_ or peptide treatment was evaluated as follows:

Cell viability (%) = A_S_/A_N_ × 100% (3)

where A_S_ and A_N_ were the absorbance of treatment and control wells, respectively.

### 3.5. Reactive Oxygen Species (ROS) Assay

Production of ROS was monitored using a ROS Assay Kit (Beyotime Biotechnology Co., Ltd., Shanghai, China). RAW 264.7 cells were cultured in 96-well plates with a density of 1 × 10^5^ cells/mL for 24 h. Cells were treated with various concentrations of peptides for 24 h, and then exposed to H_2_O_2_ (500 μmol/L) for 8 h. The experiments were implemented in the control group (not treated with peptides and H_2_O_2_), the model group (treated with 500 µM H_2_O_2_), the low-dose group (treated with 100 µg/mL peptides + 500 µM H_2_O_2_), the middle-dose group (treated with 150 µg/mL peptides + 500 µM H_2_O_2_), and the high-dose group (treated with 200 µg/mL peptides + 500 µM H_2_O_2_). RAW264.7 were incubated for 20 min in a 37 °C cell incubator with 5 µL 2′,7′- dichlorodihydrofluorescein diacetate (DCFH-DA). Cells were washed three times with serum-free cell culture medium and then observed under a fluorescence microscope.

### 3.6. Assays for Antioxidant Enzyme Activity

RAW264.7 cells (1 × 10^5^ cells/mL) were seeded in 96-well plates, and exposed to 500 µmol/L H_2_O_2_ for 8 h with or without various concentrations of peptide pre-treatment for 24 h. RAW264.7 cells were washed with Phosphate-buffered saline (PBS), and then lysed with cell lysate. The supernatants were collected following centrifugation at 2000 × *g* for 5 min at 4 °C. The activity of SOD, GSH-Px, and MDA content was determined by using corresponding diagnostic kits according to the manufacturer’s instructions (Nanjing Jiancheng Bioengineering Institute, Nanjing, China).

### 3.7. Statistical Analysis

Each experiment was carried out in triplicate. Data were presented as means and standard deviations. Results were analyzed using Microsoft Excel 2010 (Redmond, WA, USA), and significant differences (*p* < 0.05) between data were identified by Duncan’s multiple range test in the software SPSS (SPSS Inc., Chicago, IL, USA).

## 4. Conclusions

Microwave-assisted hydrolysis of collagen is a promising method for preparation of antioxidant peptides. Microwave radiation can significantly increase the content and antioxidant activity of small molecular weight peptides. The hydrolyzate fragment CP_S_ (Mw ≤ 1 kDa) obtained by microwave-assisted alkaline protease hydrolysis of collagen from sea cucumber *Acaudina molpadioides* exhibited the good scavenging activities of DPPH and OH•. CP_S_ also exhibited a significant protective effect on H_2_O_2_-injured RAW264.7 cells by promoting cell proliferation, reducing the levels of ROS and MDA, and enhancing antioxidant enzyme (SOD and GSH-Px) activity. Therefore, it is recommended, as a result of the current study, to explore CP_S_ as a potential natural antioxidant and utilize microwave-assisted hydrolysis as a method of obtaining high levels of antioxidant peptides from naturally occurring high-molecular weight protein molecules. 

## Figures and Tables

**Table 1:** Effect of microwave-assisted hydrolysis of ASC-Am on peptide content and molecular weight distribution.

Hydrolysates	Content (%)		Mw Distribution (%)	
	0 W	250 W	0 W	250 W
CP_L_ (Mw > 5 kDa)	100	90.3 ± 4.3	21.7 ± 2.1	15.7 ± 1.5
CP_M_ (1 kDa < Mw ≤ 5 kDa)	100	148.0 ± 8.5	20.3 ± 1.2	24.0 ± 1.8
CP_S_ (Mw ≤ 1 kDa)	100	130.2 ± 6.4	58.0 ± 3.1	60.3 ± 2.9
